# Biochemical aspects of bacterial strategies for handling the incomplete translation processes

**DOI:** 10.3389/fmicb.2014.00170

**Published:** 2014-04-10

**Authors:** Yoshihiro Shimizu

**Affiliations:** Laboratory for Cell-Free Protein Synthesis, Quantitative Biology Center – RIKENKobe, Hyogo, Japan

**Keywords:** tmRNA, SmpB, ArfA, RF2, ArfB, *trans*-translation, ribosome rescue, quality control

## Abstract

During protein synthesis in cells, translating ribosomes may encounter abnormal situations that lead to retention of immature peptidyl-tRNA on the ribosome due to failure of suitable termination processes. Bacterial cells handle such situations by employing three systems that rescue the stalled translation machinery. The transfer messenger RNA/small protein B (tmRNA/SmpB) system, also called the *trans*-translation system, rescues stalled ribosomes by initiating template switching from the incomplete mRNA to the short open reading frame of tmRNA, leading to the production of a protein containing a C-terminal tag that renders it susceptible to proteolysis. The ArfA/RF2 and ArfB systems rescue stalled ribosomes directly by hydrolyzing the immature peptidyl-tRNA remaining on the ribosome. Here, the biochemical aspects of these systems, as clarified by recent studies, are reviewed.

## INTRODUCTION

During cellular protein synthesis, ribosomes interact with a variety of macromolecules to enable the precise translation of genetic information into functional polypeptides. One of the most frequent ribosomal interactions is the process of sense codon decoding, in which a codon of mRNA interacts with an anticodon of tRNA in the A site of the ribosome. In bacteria, elongation factor Tu (EF-Tu) plays a crucial role in this process by delivering aminoacyl-tRNA to the ribosomal A site as part of a ternary complex (aminoacyl-tRNA/EF-Tu/GTP). When the anticodon of aminoacyl-tRNA in the ternary complex matches the mRNA codon in the ribosomal A site, GTP hydrolysis is stimulated and EF-Tu changes its conformation, leading to dissociation of the EF-Tu/GDP binary complex, accommodation of aminoacyl-tRNA at the A site of the ribosome, and transfer of a nascent polypeptide to the aminoacyl-tRNA.

Although its molecular mechanism is similar to that of sense codon decoding, termination codon decoding does not require both tRNA and EF-Tu. In this process, a termination codon of mRNA interacts with class-1 release factors (RFs) including RF1 and RF2, at the A site of the ribosome. Like the anticodon of tRNA in sense codon decoding, a specific region of class-1 RFs, known as a peptide anticodon (PAT motif for RF1 and SPF motif for RF2; Ito et al., 2000), recognizes and interacts directly with the mRNA termination codon in the decoding center of the ribosomal A site. Class-1 RFs can be accommodated at the ribosomal A site without the requirement for factors such as EF-Tu. Unlike aminoacyl-tRNA accommodated at the A site, class-1 RFs do not receive the nascent polypeptide of peptidyl-tRNA in the ribosomal P site but catalyze peptidyl-tRNA hydrolysis through a specific GGQ amino acid motif (Frolova et al., 1999; Song et al., 2000). Recent structural analyses of complexes composed of a ribosome and class-1 RFs have provided evidence for these molecular mechanisms (Korostelev et al., 2008; Laurberg et al., 2008; Korostelev, 2011).

Bacterial cells have evolved processes that enable ribosomes to progress in the absence of codon decoding. Because these processes generally take place when a ribosome is abnormally stalled by the lack of binding of a molecule to the A site, they are termed ribosome rescue processes. Ribosome stalling is most commonly caused by the lack of an in-frame termination codon in a mRNA, which can occur as a result of digestion of the mRNA (Chadani et al., 2011a; Garza-Sánchez et al., 2011), termination of transcription before the termination codon (Abo et al., 2000; Kobayashi et al., 2008), or misreading of the termination codon (Ueda et al., 2002). In these situations, the ribosome typically reaches the 3′-end of mRNA and translation is stopped by the absence of a codon in the A site. Abortive pausing of translation at certain regions of mRNAs can also occur as a result of ribosome stalling caused by the occurrence of a rare codon cluster (Roche and Sauer, 1999), amino acid starvation (Garza-Sánchez et al., 2008; Li et al., 2008), or the presence of specific sequences that cause elongation arrest (Sunohara et al., 2004). In these situations, immature peptidyl-tRNA remains on the ribosome and the resultant ribosome/mRNA/peptidyl-tRNA complex is stable enough to inhibit ribosome recycling; therefore, ribosome rescue processes are essential for its recovery.

In bacteria, the transfer messenger RNA/small protein B (tmRNA/SmpB) system (also called the *trans*-translation system), the alternative ribosome rescue factor A (ArfA)/RF2 system, and the alternative ribosome rescue factor B (ArfB) system are involved in the rescue of stalled ribosomes. This review focuses on the biochemical aspects of these three processes, as elucidated by recent studies.

## THE *TRANS*-TRANSLATION SYSTEM

**FIGURE 1:**
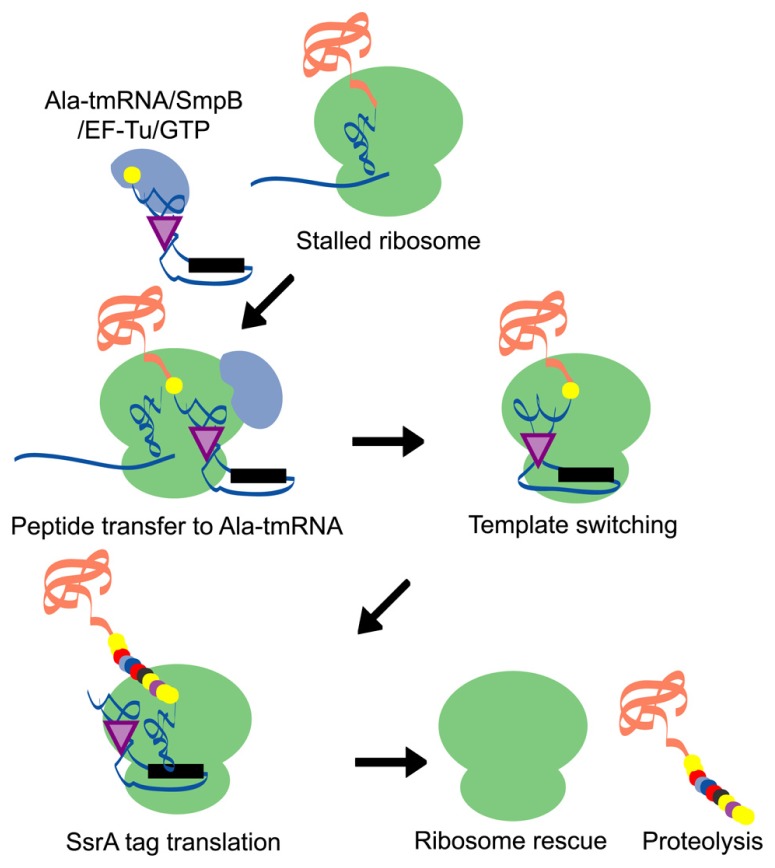
**A schematic model of the *trans*-translation system.** A complex comprised of Ala-tmRNA, SmpB, EF-Tu, and GTP enters the A site of the stalled ribosome and receives the nascent polypeptide from the peptidyl-tRNA in the ribosomal P site. The template is then switched to the ORF region of tmRNA and the SsrA tag is synthesized and attached to the C-terminus of the nascent polypeptide. Translation ceases at the termination codon in the ORF region of tmRNA and the ribosome is rescued. The SsrA-tagged nascent polypeptide is identified by the proteolysis system and degraded.

The *trans*-translation system is a bypass process for the translation machinery. When a ribosome lacks an in-frame termination codon, translation is stalled at the 3′ end of the mRNA. Subsequently, tmRNA, a unique molecule that acts as both a tRNA and an mRNA, recognizes the stalled ribosome/mRNA/peptidyl-tRNA complex and is accommodated at the ribosomal A site as alanyl-tmRNA. After A site entry, translation is switched from the incomplete mRNA to the open reading frame (ORF) of the tmRNA, which contains an authentic translation termination codon. The tmRNA-encoded peptide sequence attached to the C-terminus of newly synthesized proteins, known as an SsrA tag, acts as a degradation signal for proteolysis. Through this process, stalled ribosomes are recycled for new translation reactions and the SsrA-tagged proteins are degraded immediately, which may contribute to the maintenance of both the translation process and the quality of cellular proteins (**Figure 1**).

### THE STRUCTURE AND FUNCTION OF tmRNA

**FIGURE 2:**
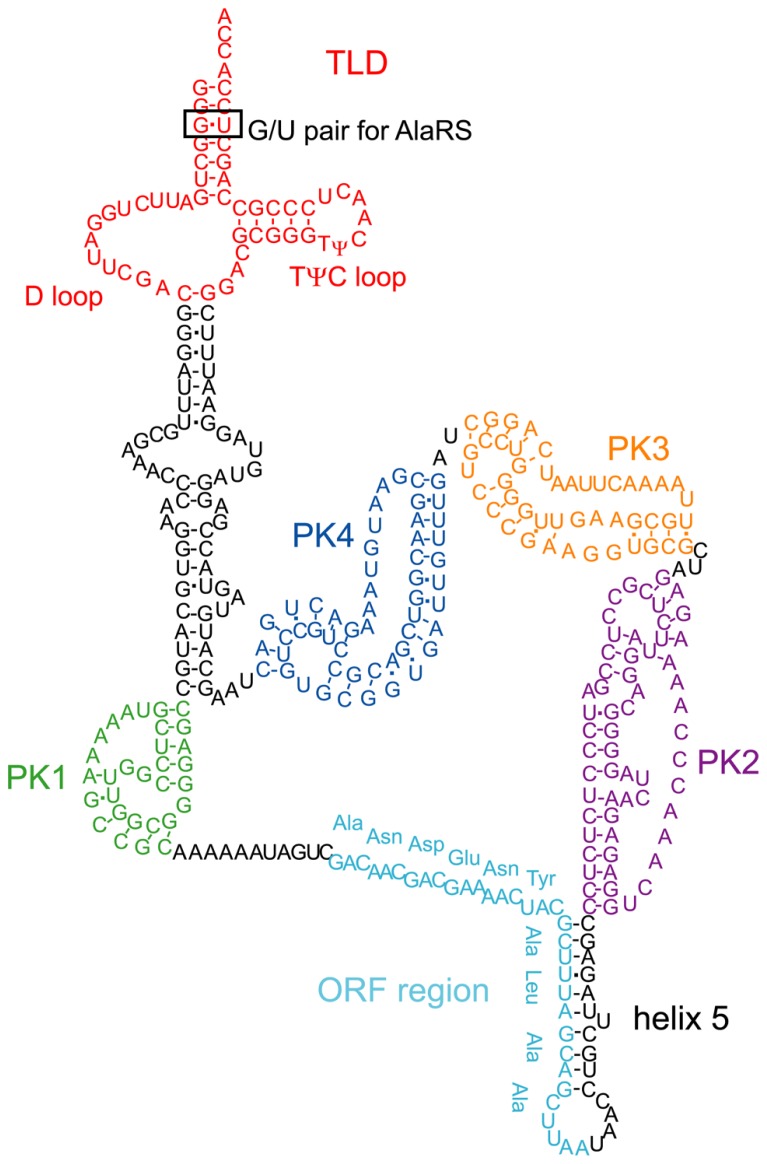
**The secondary structure of *E. coli* tmRNA.** The TLD (red), PK1 (green), ORF (light blue), helix 5, PK2 (purple), PK3 (orange), and PK4 (blue) regions are indicated. The TLD contains a D loop, a conserved TψC loop, and a G/U pair in the acceptor stem, the latter of which is recognized by alanyl-tRNA synthetase.

*Escherichia coli* tmRNA consists of 363 nucleotides and contains a tRNA-like domain (TLD), an ORF region encoding an SsrA tag, and four pseudoknot structures (PK1–4; **Figure 2**). One of the characteristic features of *E. coli* tmRNA is the TLD, in which the 5′ and 3′ terminal regions of tmRNA combine to form a tRNA-like structure. Like standard tRNA, tmRNA has an acceptor stem with a 3′-CCA end, as well as a TψC stem and loop structure that includes conserved bases. These features of tmRNA not only facilitate its recognition and processing by RNase P (Komine et al., 1994), but also allow it to accept nucleotide modifications such as thymine and pseudouridine in the TψC loop (Felden et al., 1998) in a manner similar to that of standard tRNA. Moreover, the acceptor stem region of tmRNA has a G/U wobble base pair, which is a determinant base pair in the acceptor stem of tRNA^Ala^ for recognition by alanyl-tRNA synthetase (Hou and Schimmel, 1988; Francklyn and Schimmel, 1989). Accordingly, tmRNA can undergo alanylation by alanyl-tRNA synthetase (Komine et al., 1994).

The ORF region of *E. coli* tmRNA is located downstream of the first pseudoknot structure (PK1) and encodes a peptide with the sequence ANDENYALAA (**Figure 2**). Together with the alanine residue attached by tmRNA itself, the AANDENYALAA peptide forms an SsrA degradation signal tag. The YALAA portion of the tag is important for degradation by ClpXP or ClpAP protease (Gottesman et al., 1998; Kim et al., 2000). In addition, the asparagine residue at the third position of the SsrA tag is important for recognition by SspB (stringent starvation protein B), an adaptor protein that stimulates the degradation of tagged proteins by ClpXP (Levchenko et al., 2000; Lies and Maurizi, 2008).

Other structural elements of tmRNA also affect its function. The region containing PK1 located upstream of the ORF is important for the *trans*-translation reaction (Nameki et al., 1999), although formation of the pseudoknot structure is not absolutely necessary (Tanner et al., 2006; Wower et al., 2009). Furthermore, disruption of the three pseudoknot structures (PK2–4) located downstream of the ORF and the helix 5 region that contains the partial ORF sequence also impair tmRNA function, even though these regions are not indispensable (Nameki et al., 2000; Wower et al., 2004).

### THE ADAPTER PROTEIN SmpB IS ESSENTIAL FOR tmRNA FUNCTION

The adapter protein SmpB is essential for the overall functions of tmRNA in the *trans*-translation process. SmpB was originally discovered as a protein whose gene is tandemly arranged with the *ssrA* gene (encoding tmRNA) in *E. coli* and *Salmonella typhimurium*. Deletion of the *smpB* gene in *E. coli* produces a phenotype identical to that of *ssrA*-defective cells and SmpB binds specifically to tmRNA with high affinity (Karzai et al., 1999).

NMR (nuclear magnetic resonance) analyses of SmpB from *Aquifex aeolicus* and *Thermus thermophilus* showed that the proteins consist of a globular core domain with a flexible C-terminal (C-tail) region (Dong et al., 2002; Someya et al., 2003). The core domain forms an oligonucleotide-binding fold (Murzin, 1993) that is similar to several RNA-binding proteins associated with the translation machinery, including ribosomal protein S17, initiation factor 1 (IF1), and the N-terminal domain of aspartyl-tRNA synthetase (Draper and Reynaldo, 1999).

**FIGURE 3:**
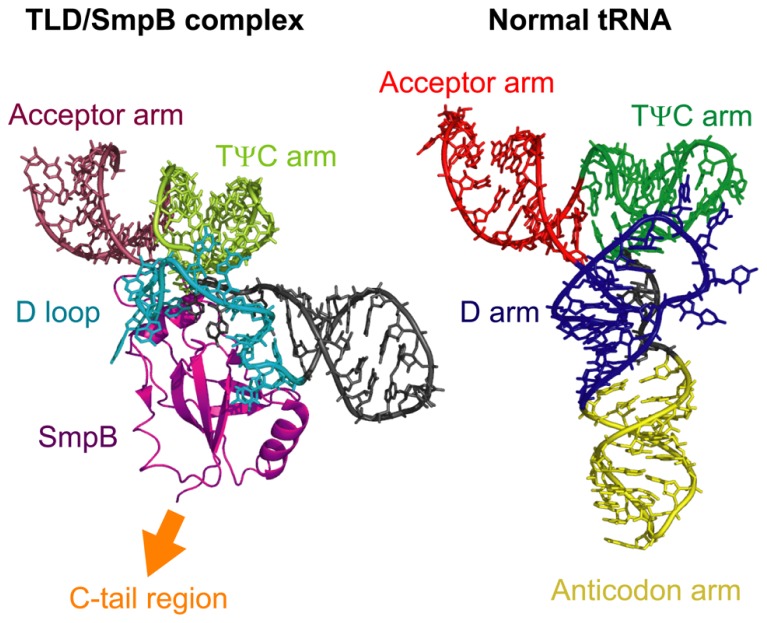
**A comparison of the structures of the TLD/SmpB complex and normal tRNA.** The left panel shows the crystal structure of the complex comprised of the TLD of tmRNA and SmpB from *Thermus thermophilus* and the right panel shows that of normal tRNA (*E. coli* tRNA^Cys^). The acceptor arm, TψC arm, D loop or D arm, and anticodon arm, or SmpB are indicated. The C-tail region of SmpB extends to the opposite side of the TLD. The structural coordinates for the TLD/SmpB and normal tRNA complexes were taken from PDB entries 2CZJ (Bessho et al., 2007) and 1B23 (Nissen et al., 1999), respectively.

The core domain and the C-tail region of SmpB play distinct roles in the *trans*-translation process. Consistent with its oligonucleotide-binding fold configuration, the core domain of SmpB plays a role in the interaction with tmRNA. In the crystal structure of the complex composed of the entire TLD of tmRNA and SmpB from *T. thermophilus* (Bessho et al., 2007), SmpB binds to the bottom of the D loop and TψC stem of tmRNA; this region corresponds to the D stem and anticodon arm of the L-shaped tRNA (**Figure 3**). Binding of SmpB to the TLD of tmRNA contributes to the formation of an L-shaped structure by compensating for the lack of a D stem in tmRNA and thereby assisting D and TψC arm interaction, and by stabilizing the coaxial structure of the TψC and acceptor stems (Bessho et al., 2007). Thus, the core domain of SmpB and the TLD of tmRNA cooperatively mimic canonical tRNA in a sophisticated manner (**Figure 3**). SmpB enhances alanylation of tmRNA or the TLD by alanyl-tRNA synthetase (Barends et al., 2001; Hanawa-Suetsugu et al., 2002; Shimizu and Ueda, 2002, 2006) and promotes protection of the aminoacyl moiety of alanyl-TLD by EF-Tu (Shimizu and Ueda, 2006). Stabilization of the acceptor arm region by SmpB may contribute to these effects because both alanyl-tRNA synthetase and EF-Tu interact with the acceptor arm of tRNA (Nissen et al., 1995; Swairjo et al., 2004).

In the crystal structure of the tmRNA/SmpB complex, the C-tail region of SmpB extends to the opposite side of the TLD and is positioned in a proximal region corresponding to the anticodon loop of canonical L-shaped tRNAs (**Figure 3**). This arrangement locates the C-tail region of SmpB close to the decoding region of the 30S ribosomal subunit during entry of tmRNA into the ribosomal A site (Kaur et al., 2006). Furthermore, deletion of more than seven amino acids from the C-terminus of this region inhibits the addition of the SsrA tag to the nascent polypeptide *in vivo* (Jacob et al., 2005; Sundermeier et al., 2005) or alanyl-transfer to the nascent polypeptide on the ribosome *in vitro* (Shimizu and Ueda, 2006), without affecting the alanylation efficiency of tmRNA. In addition, several mutations in the conserved residues of the C-tail region have similar inhibitory effects (Sundermeier et al., 2005; Miller et al., 2011). These studies indicate that the C-tail region of SmpB plays a crucial role in the *trans*-translation process through an interaction with the decoding region of the 30S ribosomal subunit.

### THE *TRANS*-TRANSLATION PROCESS ON THE RIBOSOME

**FIGURE 4:**
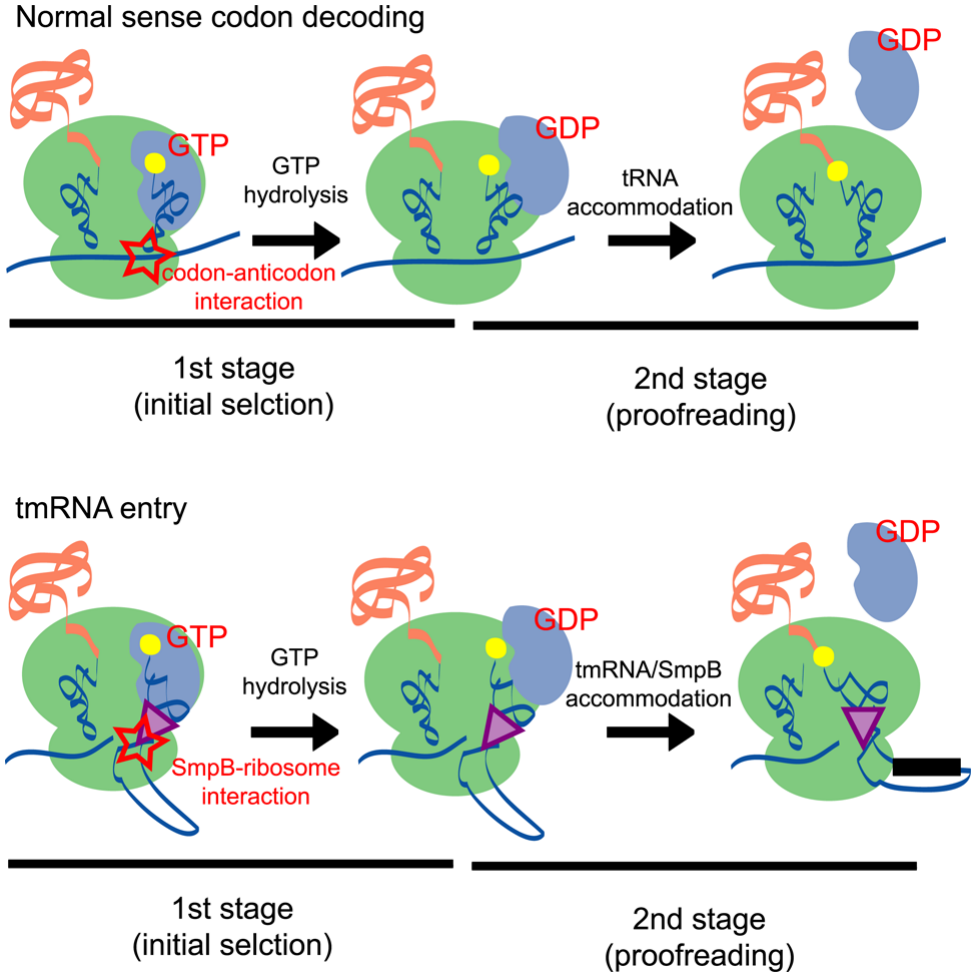
**A schematic comparison of the normal sense codon decoding (upper panel) and tmRNA entry (lower panel) processes.** In the first stage (initial selection) of the normal sense codon decoding process, the codon–anticodon interaction stimulates GTP hydrolysis and induces a conformational change of EF-Tu. By contrast, the SmpB-ribosome interaction may stimulate GTP hydrolysis of EF-Tu in the tmRNA entry process. In the second stage (proofreading) of the processes, aminoacyl-tRNA is accommodated at the A site of the ribosome according to the codon–anticodon interaction, whereas the tmRNA/SmpB complex is accommodated at the A site presumably according to an interaction between SmpB and the ribosome.

During the *trans*-translation process, the alanyl-tmRNA/SmpB complex can enter the A site of the ribosome without the requirement for codon–anticodon interaction. The mechanisms involved in sense codon decoding in the canonical translation system have been well-characterized by kinetic experiments (Rodnina and Wintermeyer, 2001; Daviter et al., 2006), cryo-electron microscopic analyses (Stark et al., 2002; Valle et al., 2002, 2003a; Schuette et al., 2009; Villa et al., 2009), X-ray crystallographic analyses (Schmeing et al., 2009; Voorhees et al., 2010), and single-molecule observations (Blanchard et al., 2004; Lee et al., 2007; Geggier et al., 2010). These studies have identified a number of intermediate states of the sense codon decoding complex and have demonstrated that the selection of cognate aminoacyl-tRNA is achieved in two stages that are separated by irreversible GTP hydrolysis (**Figure 4**).

In this scheme, codon–anticodon interactions play a critical role in both the first and second stages of the sense codon decoding process. In the first stage, codon–anticodon interaction in the A site induces structural rearrangements of the 30S ribosomal subunit; formation of the first and second base pairs expose the A1492 and A1493 residues from helix 44 of 16S rRNA and the G530 residue of 16S rRNA also undergoes a conformational change that enables it to interact with the second and third base pairs (Ogle et al., 2001). Moreover, conformation of the 30S subunit is changed from an open to a closed form upon codon–anticodon binding (Ogle et al., 2002). These observations suggest that structural rearrangements at the decoding site are essential features of tRNA selection. It should be noted that similar structural rearrangements are also observed in the ribosome retaining near-cognate tRNA at the A site (Demeshkina et al., 2012). However, in this case, codon–anticodon interaction is destabilized and causes dissociation of tRNA from the ribosome. The stabilization of the codon–anticodon interaction in the ribosomal A site may promote a kinked conformation of the tRNA structure and destabilize the interaction between the 3′ end of aminoacyl-tRNA and EF-Tu, thereby triggering GTP hydrolysis (Frank et al., 2005; Schmeing et al., 2009). In the second stage of the sense codon decoding process, codon–anticodon interactions support the so-called molecular spring movement of aminoacyl-tRNA that is released from its high-energy state (Frank et al., 2005; Sanbonmatsu et al., 2005; Whitford et al., 2010).

In contrast to the sense codon decoding process, codon–anticodon interactions do not occur during tmRNA/SmpB entry because tmRNA does not possess an anticodon and the ribosome does not contain mRNA codon in its A site. Despite this situation, the tmRNA/SmpB complex binds to and facilitates the hydrolysis of EF-Tu (Barends et al., 2001; Valle et al., 2003b; Kaur et al., 2006) to enable its entry into the ribosomal A site (Shimizu and Ueda, 2006). Furthermore, the C-tail region of SmpB plays a crucial role in this process through an interaction with the decoding region of the 30S ribosomal subunit, suggesting that the interaction between the ribosome and the C-tail region of SmpB compensates for the absent codon–anticodon interaction.

The molecular mechanism of the compensation by SmpB has been revealed in part by the recently reported crystal structure of the complex composed of the *T. thermophilus* ribosome, a partial fragment of tmRNA, SmpB, and EF-Tu, which was trapped in the GDP form after GTP hydrolysis using the antibiotic kirromycin (Neubauer et al., 2012). In this structure, the C-tail region of SmpB forms an α-helix as predicted (Miller et al., 2011); this region extends toward the mRNA path downstream of the A site to interact with the ribosomal protein S5. The structural rearrangements are similar with that of the ribosomal complex involved in canonical sense codon decoding, suggesting that binding of SmpB to the decoding region of the 30S ribosomal subunit induces similar structural rearrangements in the ribosome, which may be necessary for the stimulation of GTP hydrolysis.

However, the exact mechanisms involved in the stimulation of GTP hydrolysis during tmRNA/SmpB entry may not be identical to the process that occurs during sense codon decoding. For example, mutations of the G530, A1492, and A1493 residues of 16S rRNA, which reduce the rates of peptidyl transfer and GTP hydrolysis in canonical decoding drastically, do not affect these reactions during the rescue of stalled ribosomes by tmRNA (Miller et al., 2011). In addition, the ribosomal protein S12, which has a stabilizing effect during canonical decoding, does not interact with SmpB (Neubauer et al., 2012). These findings may indicate that the interactions between the ribosome and the codon–anticodon base pairs that occur during canonical decoding are not essential for structural rearrangements of the 30S ribosomal subunit and subsequent GTP hydrolysis. SmpB may use a different mechanism to compensate for the lack of codon–anticodon interaction in the A site of the ribosome to stimulate GTP hydrolysis.

Intriguingly, peptidyl-transfer rates are reduced markedly when the C-tail region of SmpB is mutated or deleted, although GTP hydrolysis rates are not affected (Miller et al., 2011). This finding suggests that the C-tail region may play a role after GTP hydrolysis, presumably in the step corresponding to the second proofreading stage in the canonical decoding process (**Figure 4**). Considering that key residues responsible for controlling the peptidyl-transfer rate are dispersed in the entire region of the C-tail (Sundermeier et al., 2005; Miller et al., 2011), interactions between these residues and several regions of the ribosome may play important roles in this process.

A number of questions related to the dynamics of accommodation of the tmRNA/SmpB complex into the ribosomal A site during the *trans*-translation process remain unanswered. For example, the mechanisms by which the ORF region of tmRNA is loaded into the A site and the resume codon in the ORF region is determined are still unknown. Although cryo-electron microscopic visualizations of the ribosome structure after translocation of the tmRNA to the P site (Fu et al., 2010; Weis et al., 2010; Ramrath et al., 2012) and mutational studies of both tmRNA and SmpB (Williams et al., 1999; Lee et al., 2001; Miller et al., 2008; Watts et al., 2009; Camenares et al., 2013) have added some insights, additional biochemical, structural, single-molecule imaging (Zhou et al., 2011), and simulative studies (Sanbonmatsu et al., 2005) are necessary to elucidate the precise dynamics of the *trans*-translation system on the ribosome.

## TWO ALTERNATIVE SYSTEMS FOR RIBOSOME RESCUE

The *trans*-translation system has long been regarded as an exclusive method of ribosome rescue in bacteria. However, although it is essential for the viability of several phylogenetic groups (Huang et al., 2000; Glass et al., 2006; Ramadoss et al., 2013a), other groups, including *E. coli*, do not require the *trans*-translation system for survival (Komine et al., 1994; Karzai et al., 1999; Wiegert and Schumann, 2001), suggesting the existence of alternative pathways for stalled ribosome rescue. In fact, two alternative systems were recently identified: the ArfA/RF2 system and the ArfB system. The ribosome rescue mechanisms of these two pathways are described in the following sections.

### THE ArfA/RF2 SYSTEM

ArfA (previously called YhdL) was originally identified as a protein factor essential for the viability of *E. coli* in the absence of the *trans*-translation system (Chadani et al., 2010). Using genetic screening, Chadani et al. (2010) determined that the *arfA* gene is required for suppression of the growth defect of cells lacking this system and showed that double depletion of ArfA and tmRNA from cells causes a loss of viability, indicating that ArfA plays a role complementary to that of the *trans*-translation system.

The *E. coli arfA* gene encodes a small protein of 72 amino acids. A conserved hairpin structure within the *arfA* mRNA (Schaub et al., 2012) is a target of RNase III and the ArfA protein is expressed from the truncated mRNA lacking an in-frame termination codon. Although the full-length ArfA polypeptide cannot be expressed in normal cells, the truncated form containing 53–55 of the N-terminal residues retains full functionality (Chadani et al., 2011a; Garza-Sánchez et al., 2011). When the *trans*-translation system is active in cells, the ribosome translating the *arfA* mRNA becomes a target of tmRNA/SmpB and the SsrA tag is attached to the synthesized ArfA peptide, leading to its subsequent degradation by SspB/ClpXP. Therefore, ArfA expression is regulated by the *trans*-translation system and may function as a backup system that substitutes for a deficient or suppressed *trans*-translation system.

**FIGURE 5:**
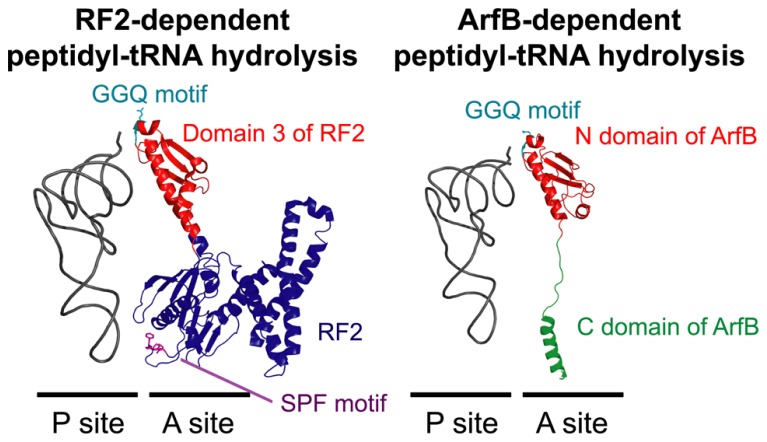
**A structural comparison of RF2-dependent (left panel) and ArfB-dependent (right panel) peptidyl-tRNA hydrolysis on the ribosome.** In the normal translation process, RF2 binds to the ribosomal A site according to the interaction between the termination codon and the SPF motif (purple) in RF2. In the ArfA/RF2 ribosome rescue process, RF2 may bind to the A site similarly, albeit in a codon-independent and ArfA-dependent manner. ArfB binds to the A site according to the interaction between the C domain of ArfB (green) and the ribosome in a codon-independent manner. In both reactions, the hydrolysis of peptidyl-tRNA in the P site is catalyzed by a specific GGQ amino acid motif (light blue) in the conserved domain of RF2 (domain 3; red) or ArfB (N domain; red). The structural coordinates of the RF2-dependent and ArfB-dependent hydrolysis complexes were taken from PDB entries 3F1E (Korostelev et al., 2008) and 4DH9 (Gagnon et al., 2012), respectively.

Although ArfA can bind to the 50S subunit of the ribosome and peptidyl-tRNA in the ribosomal P site can be hydrolyzed in cell-free protein synthesis reaction mixtures containing S30 cell extract (Chadani et al., 2010), ArfA alone cannot hydrolyze peptidyl-tRNA on the purified ribosome complex, indicating the existence of factors that function cooperatively (Chadani et al., 2011b). Recent studies using a reconstituted cell-free protein synthesis system (Shimizu et al., 2001) showed that RF2, which catalyzes peptidyl-tRNA hydrolysis in the canonical termination step (Frolova et al., 1999; Song et al., 2000), also plays a catalytic role in peptidyl-tRNA hydrolysis during ArfA-dependent rescue of stalled ribosomes (Chadani et al., 2012; Shimizu, 2012). Mutation of the GGQ amino acid motif in RF2 disrupts this process (Chadani et al., 2012). RF2 can bind to stalled ribosomes in the presence of ArfA, whereas RF1 is unable to bind to ribosomes in the presence or absence of ArfA (Shimizu, 2012), indicating that ArfA recruits RF2 but not RF1 into the A site of the stalled ribosome, where it promotes peptidyl-tRNA hydrolysis (**Figure 5**).

The mechanism by which ArfA recruits RF2 into the stalled ribosome is still unknown. A18T-mutated ArfA, which suppresses ArfA-dependent ribosome rescue (Chadani et al., 2010), is unable to support peptidyl-tRNA hydrolysis *in vitro*. However, this mutation does not inhibit binding of RF2 to the ribosome (Shimizu, 2012), suggesting that simply recruiting RF2 into the stalled ribosome is not sufficient for the ArfA-dependent ribosome rescue system. The mechanism by which ArfA selectively recruits RF2 but not RF1 is also intriguing because the primary sequences and structures of RF1 and RF2 on the ribosome are very similar (Laurberg et al., 2008; Korostelev et al., 2008; Korostelev, 2011). Elucidating these mechanisms will contribute to understanding the protein synthesis system and the basis for bacterial evolution, and may also be useful for antibiotic development (Ramadoss et al., 2013b) because neither the *trans*-translation system nor the ArfA systems are found in animals.

### THE ArfB SYSTEM

Evidence that ArfB (previously called YaeJ) plays a role in stalled ribosome rescue was reported independently by two research groups (Chadani et al., 2011b; Handa et al., 2011). Handa et al. (2011) demonstrated that ArfB hydrolyzes peptidyl-tRNA on the ribosome *in vitro* and Chadani et al. (2011b) genetically identified ArfB as a multicopy suppressor of the growth defect of *ssrA*/*arfA* double mutants and then showed similar results to those reported by Handa et al. (2011). Notably, immature colon carcinoma transcript 1 (ICT1), an ArfB homolog present in the human mitochondrial ribosome complex, also displays ribosome-dependent peptidyl-tRNA hydrolysis activity (Richter et al., 2010), suggesting that ICT1 plays a role in stalled ribosome rescue in human mitochondria.

Compared with the *trans*-translation and ArfA/RF2 systems, the molecular mechanism of ArfB-dependent ribosome rescue is relatively simple. ArfB consists of a C-terminal unstructured region and a globular N-terminal domain containing a GGQ amino acid motif that resembles domain 3 of class-1 RFs. Analysis of the crystal structure of *E. coli* ArfB bound to the *T. thermophilus* ribosome with the initiator tRNA^fMet^ (Gagnon et al., 2012) showed that the N-terminal domain is located in the A site of the 50S ribosomal subunit and the GGQ motif is positioned in the peptidyl-transferase center adjacent to the CCA end of the initiator tRNA^fMet^ in the ribosomal P site. Together with the finding that mutation of the GGQ motif in ArfB inhibits its peptidyl-tRNA hydrolysis activity (Chadani et al., 2011b; Handa et al., 2011), the structure indicates that this motif is responsible for peptidyl-tRNA hydrolysis to rescue stalled ribosomes.

In addition to mutation of the N-terminal domain, truncation of the C-terminal unstructured region of ArfB also suppresses peptidyl-tRNA hydrolysis on the ribosome (Chadani et al., 2011b; Handa et al., 2011). Analysis of the crystal structure showed that, similar to the C-tail region of SmpB in the tmRNA entry process of the *trans*-translation system (Neubauer et al., 2012), the C-terminal unstructured region of ArfB forms an α-helix and is accommodated inside the mRNA path downstream of the A site (Gagnon et al., 2012). Several point mutations of basic residues in the C-terminal region suppress the activity of ArfB *in vitro*, indicating that the interaction between the mRNA path and these residues plays an important role in ArfB-mediated ribosome rescue (Kogure et al., 2014). In summary, ArfB plays an independent role in stalled ribosome rescue by interacting with the A site of the ribosome and catalyzing the hydrolysis of peptidyl-tRNA in the P site in a codon–independent manner (**Figure 5**).

## ESSENTIAL FEATURES OF THE RIBOSOMAL SITUATION REQUIRED FOR PROPER FUNCTIONING OF THE THREE RESCUE SYSTEMS

A common feature of the three ribosome rescue pathways described here is that they may be structurally designed to selectively target stalled ribosomes lacking a codon in the A site. The C-tail regions of SmpB and ArfB are both accommodated in the mRNA path downstream of the ribosomal A site (Gagnon et al., 2012; Neubauer et al., 2012), suggesting that binding of these proteins is inhibited if an mRNA is located downstream of the A site, which is a common feature of translating ribosomes. The structure of the ribosome complex containing ArfA and RF2 has not yet been clarified; however, since the ArfA/RF2 system uses RF2 for peptidyl-tRNA hydrolysis, binding of RF2 to the A site may be limited to ribosomes in which the A site is either unoccupied or occupied by a UAA or UGA codon, which matches the peptide anticodon region (SPF motif) of RF2 (Ito et al., 2000).

Accordingly, *in vitro* studies showed that the presence of an mRNA downstream of the A site inhibits the three ribosome rescue pathways. An *in vitro* study of the *trans*-translation system showed that the rate of alanyl-transfer to the nascent polypeptide is inversely proportional to the length of the mRNA downstream of the A site (Ivanova et al., 2004). Furthermore, ribosome rescue by the ArfA/RF2 system is reduced significantly if the stalled ribosome contains additional mRNA sequences downstream of the A site (Shimizu, 2012). The ribosome rescue activity of the ArfB system is also inversely proportional to the length of mRNA downstream of the A site, although it is maintained when a sufficient length of mRNA, which fully covers the mRNA path downstream of the A site on the ribosome, remains on the ribosome (Shimizu, 2012). The fact that ArfB is able to promote ribosome rescue in the presence of a sufficient length of mRNA is in agreement with the finding that ArfB can hydrolyze peptidyl-tRNA on ribosomes stalled by a rare codon cluster *in vitro* (Handa et al., 2011). These studies may suggest that the flexibility of mRNA allows the C-terminal region of ArfB to bind to the ribosome by avoiding a steric clash (Gagnon et al., 2012).

The *in vitro* studies described above suggest that the ArfB system may interfere with the normal translation system because it can cover a wider range of ribosomal situations than the *trans*-translation or ArfA/RF2 systems. There is currently no explanation for the broader specificity of the ArfB system. Notably, endogenous ArfB cannot restore the viability of *E. coli* cells depleted of both ArfA and tmRNA, whereas overexpression of exogenous ArfB does restore this defect (Chadani et al., 2011b). This lack of compensation may be due to the lower expression level of ArfB compared to SmpB in *E. coli* (about 0.5 and 15 protein molecules/cell, respectively; Taniguchi et al., 2010), suggesting that ArfB may not play a crucial role in ribosome rescue in *E. coli* cells. However, the situation may be different in other bacterial cells because only a subset of β- and γ-proteobacteria contains ArfA homologs (Schaub et al., 2012). The tight regulation of ArfA expression by the *trans*-translation system suggests that these two systems have complementary roles in ribosome rescue (Chadani et al., 2011a; Garza-Sánchez et al., 2011), perhaps indicating that they play major roles in the ribosome rescue process in *E. coli* cells at least. Therefore, it is reasonable to speculate that the *trans*-translation and ArfA/RF2 systems are structurally designed to target stalled ribosomes more precisely.

*In vivo* studies showed that the *trans*-translation system targets ribosomes that are stalled at certain regions of mRNA due to amino acid starvation (Garza-Sánchez et al., 2008; Li et al., 2008) or the presence of a rare codon cluster (Roche and Sauer, 1999) or specific sequence that causes elongation arrest (Sunohara et al., 2004), in which a sufficient length of mRNA may remain on the ribosome downstream of the A site. These observations may not contradict the *in vitro* studies because such pausing reportedly induces cleavage of the A site mRNA codon (Hayes and Sauer, 2003; Yamamoto et al., 2003; Sunohara et al., 2004; Garza-Sánchez et al., 2008). The mechanisms involved in this cleavage process are largely unknown, although RNase II exonuclease activity has been implicated (Garza-Sánchez et al., 2009). Additional studies of the ways in which cells handle the mRNA portion of stalled ribosomes by controlling RNase activity are necessary to understand the essential features of the ribosomal situation required for proper functioning of the rescue systems.

## Conflict of Interest Statement

The author declares that the research was conducted in the absence of any commercial or financial relationships that could be construed as a potential conflict of interest.
